# Positional Differences in the Most Demanding Scenarios of External Load Variables in Elite Futsal Matches

**DOI:** 10.3389/fpsyg.2021.625126

**Published:** 2021-02-12

**Authors:** Jordi Illa, Daniel Fernandez, Xavier Reche, Fabio R. Serpiello

**Affiliations:** ^1^Sports Performance Area, Sport Science Department, Futbol Club Barcelona, Barcelona, Spain; ^2^Institute for Health and Sport (IHES), Victoria University, Melbourne, VIC, Australia

**Keywords:** most demanding scenarios, team sport, game analysis, ultra-wideband, futsal, player monitoring, physical demands

## Abstract

The aims of this study were to analyze the peak physical demands in elite futsal by quantifying the most demanding scenarios of match play and to identify the differences between playing positions (defenders, wingers, and pivots) and the seasonal trend for five different rolling average time windows (30, 60, 120, 180, and 300 s). The most demanding scenarios of external load from distance, speed, acceleration, and deceleration variables were obtained from 14 elite futsal players using a local positioning system during 15 official matches in the premier Spanish Futsal League (2018–2019 season). The results showed an extremely large effect of the time window for all dependent variables in all positional groups. Another important finding of this study was that, in regard to the seasonal trend, only defenders reported clear moderate-large positive trends for high-speed running (>18 km⋅h^–1^) efforts, high-acceleration efforts, and high-deceleration efforts. Finally, moderate-large individual differences in player means for all dependent variables and clear differences between games for most dependent variables were found, suggesting how likely contextual factors may exert an influence on how “demanding” the most demanding scenarios are. The findings of this study provide coaches and strength and conditioning coaches further knowledge of the peak physical demands in elite futsal competition. This valuable information may lead to a more precise position-specific training prescription.

## Introduction

Futsal is an intermittent high-intensity indoor team sport involving short, high-intensity actions such as accelerations, decelerations, changes of direction, and sprints with short recovery time between efforts (Yeemin et al., 2016). Competitive matches consist of two 20-min periods characterized by the stoppage of the clock when the ball is out of play resulting in matches usually lasting 70–85% longer than the scheduled 40 min (Barbero-Álvarez et al., 2008). According to its rules, the number of substitutions during futsal matches is unlimited. As in other multidirectional outdoor and indoor team sports, an accurate understanding of the physical demands that players have to face during competition has become increasingly important to inform training program prescription (Gabbett et al., 2012) and periodization to enhance individual and team performance (Akenhead et al., 2016; Aoki et al., 2017) while reducing their susceptibility to non-functional overreaching and likelihood of injury (Bourdon et al., 2017; Fox et al., 2017; Vanrenterghem et al., 2017). Existing research has shown that throughout a competitive season, lower injury rates lead to an increased training and match-play availability of the players which have been associated with improved performance in European Soccer Leagues, International European Cups (Hägglund et al., 2013), and Qatari Professional Football (Eirale et al., 2013).

With the recent availability of wearable microsensor technology and in particular ultra-wideband (UWB) positioning systems in indoor team sports (Serpiello et al., 2018), practitioners have been provided with quantitative data to detect and measure sport-specific movements leading to a greater understanding of the external load demands of the competition (Chambers et al., 2015). Regardless of the team-sport analyzed, most of the studies that focused on defining game activity profiles have used the average of the mean activity performed by the players during competition games (Póvoas et al., 2014; Fox et al., 2018; García et al., 2020; Serrano et al., 2020) without considering the peak physical demands of the competition. For example, in a recent study conducted by Serrano et al., 2020, the influence of the match half and playing position on physical requirements in the Spanish Professional Futsal League was analyzed. Results showed that futsal defenders, pivots, and wingers averaged a relative distance covered per minute of 91 ± 9, 86 ± 6, and 95 ± 10 m.min^–1^, respectively, during the first half and 92 ± 12, 86 ± 9, and 92 ± 9 m.min^–1^, respectively, during the second half. Apparently, these results show some harmony with the playing positions’ roles with the pivots being commonly used as a target player during the offensive phase of the game, the defenders commonly marking the pivots in their own territory and wingers performing supporting defensive and offensive runs during the offensive and defensive phases of the game (Ohmuro et al., 2020). Despite this valuable information, this approach may underestimate the most demanding scenarios (MDS) of match play, also referred to as “worst-case scenarios” (Reardon et al., 2017; Cunningham et al., 2018), “most demanding passages” (Martín-García et al., 2018, 2019, 2020; Fernández et al., 2020), and “most intense periods” (Di Mascio and Bradley, 2013).

The MDS have been object of recent investigation in different team sports such as rugby union (Reardon et al., 2017; Cunningham et al., 2018), soccer (Duthie et al., 2018; Martín-García et al., 2018), basketball (Ade et al., 2016; Salazar and Castellano, 2019; Alonso et al., 2020; Fox et al., 2020; Vázquez-Guerrero et al., 2020), and rink hockey (Fernández et al., 2020). To the authors knowledge however, no research has investigated the MDS in futsal.

When analyzing the MDS for any external load variable, researchers have used rolling averages for different time epochs (ranging from 30 s to 10 min) (Whitehead et al., 2018) in an attempt to identify the most appropriate training input when designing and developing training drills (Delaney et al., 2017; Vázquez-Guerrero et al., 2020). Also important from a training prescription standpoint is that MDS have been reported to be position-dependent in soccer (Martín-García et al., 2018) and in basketball (Alonso et al., 2020; Fox et al., 2020; Vázquez-Guerrero et al., 2020).

With all this in mind, the aims of the present study were to (1) examine the peak physical demands during elite futsal competition by quantifying the MDS of match play of different relative (per minute) external load variables and (2) identify the differences between playing positions, between five different time windows and the seasonal trend in the MDS of match play for the different external load variables analyzed.

## Materials and Methods

### Design

A retrospective observational study was undertaken to quantify and analyze the positional differences of the MDS of external load of elite futsal players during 15 official matches of the premier Spanish Futsal League Liga Nacional de Fútbol Sala (LNFS) during the 2018–2019 season. An UWB electronic performance tracking system (WIMU PRO^TM^, Realtrack Systems, Almeria, Spain) was used to monitor and collect the external load data.

### Subjects

Fourteen professional futsal players (age: 28.8 ± 2.4 years, weight: 73.7 ± 6.2 kg, height: 175.9 ± 5.9 cm) from a Spanish elite team that competes in the LNFS and in the Union of European Football Associations (UEFA) Futsal Champions League were monitored using a local positioning system (WIMU Pro, Almeria, Spain). The players were categorized according to their playing positions into defenders (*n* = 4), wingers (*n* = 8), and pivots (*n* = 2). Goalkeepers were not included in this study. Players that played less than 5 minutes during the match or did not complete the match due to an injury were excluded from this study. A total of 143 observations (31 from defenders, 27 from pivots, and 85 from wingers) were collected. All players were routinely monitored throughout the course of the season and were informed of the purpose of the study and provided their written consent before the study was conducted. The experimental procedures used in this study were in accordance with the Declaration of Helsinki and were approved by the local Ethics and Scientific Committee.

### Procedures

All the matches were completed on the same official sport-specific indoor court in similar environmental conditions and played during in-season weeks, after a standard 30-min warm-up consisting of dynamic mobility and individual sport-specific skills such as passing, dribbling, and shooting. Although players were continuously monitored during warm-ups and total match time, the MDS were only analyzed when players were competing on court. All data while the players were resting after substitutions and inactivity time between periods were removed.

Data collection was carried out with a local positioning system (WIMU PRO^TM^, Realtrack Systems SL) and its corresponding software (SPRO^TM^, Realtrack Systems SL, version 946). The devices were placed in the upper part of the back, in tight-fitting harnesses. The WIMU PRO^TM^ is equipped with four 3D accelerometers (full-scale out output ranges are ± 16 g, ± 16 g, ± 32 g, and ± 400 g; 100 Hz sample frequency), three gyroscopes (8000°/s full-scale out output range; 100 Hz sample frequency), a 3D magnetometer (100 Hz sample frequency), a global positioning system (GPS; 10 Hz sample frequency), and a UWB (18 Hz sample frequency). The UWB system was installed on the court as follows: 6 antennae with UWB technology were fixed 5 m from the court perimeter line. Recently, the WIMU PRO system presented a high intra-class correlation coefficient (ICC) value for the *x*-coordinate (0.65), a very high one for the *y*-coordinate (0.85) and a good technical error of measurement: 2% (Bastida-Castillo et al., 2019).

All the variables selected to describe the external load scenarios were relative to minute. A total of seven variables were analyzed: total relative distance covered (m⋅min^–1^), high-speed running (HSR) distance (distance covered above 18 km⋅h^–1^; m⋅min^–1^), HSR efforts (number of efforts above 18 km⋅h^–1^; n⋅min^–1^), high-intensity accelerations efforts (>2 m⋅s^–2^; n⋅min^–1^), high-intensity decelerations efforts (<-2 m⋅s^–2^; n⋅min^–1^), high-intensity accelerations distance (>2 m⋅s^–2^; m⋅min^–1^) and high-intensity decelerations distance (<-2 m⋅s^–2^; m⋅min^–1^). A moving average method was used to evaluate the MDS for five different time windows (30, 60, 120, 180, and 300 s), the most used in the bibliography (Whitehead et al., 2018) and the most common time duration on court (the 300-s window) of the players in the analyzed team. The final output database was the maximum point of the rolling average of each game play, for each variable, player, and time window.

### Statistical Analysis

To assess the effect of time windows and playing positions, a general mixed linear model was created with Proc Mixed in SAS Studio (University edition, version 9.4, SAS Institute, Cary, NC, United States). The log of each performance indicator was the dependent variable. The fixed effects were: player position (three levels: defender, pivot, and winger), the interaction of player position with the log of the time window (to estimate separate linear effects for each position), and the interaction of player position with the week of the season (to estimate separate linear seasonal trends for each position). The random effects were: player identity (14 levels) and its interaction with the log of the time window (to estimate individual differences in true player means and slopes), with an unstructured covariance matrix to allow their correlation; the game date (15 levels, to allow for differences in game means); and the residuals (with a difference variance allowed for each time window). The seasonal trend was calculated as the predicted mean at the end of the season minus the predicted mean at the beginning of the season. The magnitude of the effect of the time window was calculated as the predicted mean for the 30-s window minus the predicted mean for the 300-s window. Effects and standard deviations were back-transformed to percent units. Differences between playing position groups within each time window were not assessed due to the limited sample size. Uncertainty in the estimates of effects is presented as 90% compatibility limits. Probabilistic decisions about true (infinite-sample) magnitudes accounting for the uncertainty were based on one-sided hypothesis tests of substantial magnitudes (Lakens et al., 2018). The *p*-value for rejecting a hypothesis of a given magnitude was the area of the sampling *t* distribution of the effect statistic with values of that magnitude. Hypotheses of substantial decrease and increase were rejected if their respective *p*-values were less than 0.05. If one hypothesis was rejected, the *p*-value for the other hypothesis was interpreted as evidence *for* that hypothesis, since the *p*-value corresponds to the posterior probability of the magnitude of the true effect in a reference Bayesian analysis with a minimally informative prior (Hopkins and Batterham, 2018, 2019). The *p*-value is reported qualitatively using the following scale: 0.25–0.75, possibly; 0.75–0.95, likely; 0.95–0.995, very likely; > 0.995, most likely (Hopkins et al., 2009). If neither hypothesis was rejected, the magnitude of the effect was considered to be unclear. Effects with sufficient probability of a magnitude (at least very likely) were deemed clear.

## Results

Descriptive statistics of the raw data for all time windows and playing positions are reported in Table 1. Overall, the analyzed time windows for the MDS of all variables displayed the typical trend of a power law which has been previously identified in similar research studies in other sports (Duthie et al., 2018). There was a clear, extremely large effect of the time window for all dependent variables in all positional groups (Table 2). The analysis of the seasonal trend (i.e., as the predicted mean of each variable at the end of the season minus the predicted mean at the beginning of the season) returned varied results, with some unclear effects and some clear effects of moderate to large magnitudes (Table 2).

**TABLE 1:** Descriptive statistics for the most demanding scenarios of each dependent variable assessed across five time windows.

		Time window (s)				
		30	60	120	180	300
Relative distance (m.min^–1^)	Defenders	186 ± 14	155 ± 10	133 ± 8	123 ± 7	112 ± 9
	Pivots	175 ± 15	143 ± 12	124 ± 11	115 ± 10	105 ± 9
	Wingers	187 ± 17	154 ± 14	132 ± 11	122 ± 10	113 ± 8
Distance HSR (m.min^–1^)	Defenders	47 ± 14	27 ± 8	16 ± 5	12 ± 4	8 ± 3
	Pivots	43 ± 14	24 ± 7	15 ± 6	10 ± 4	7 ± 3
	Wingers	54 ± 17	32 ± 11	20 ± 7	15 ± 5	11 ± 4
HSR efforts (n.min^–1^)	Defenders	4.5 ± 1.4	2.8 ± 0.8	1.7 ± 0.5	1.4 ± 0.4	1.0 ± 0.3
	Pivots	3.9 ± 1.4	2.4 ± 0.8	1.4 ± 0.5	1.1 ± 0.4	0.8 ± 0.3
	Wingers	4.8 ± 1.4	3.0 ± 0.9	1.9 ± 0.7	1.5 ± 0.5	1.1 ± 0.4
High accelerations distance (m.min^–1^)	Defenders	75 ± 13	52 ± 8	39 ± 6	34 ± 5	28 ± 4
	Pivots	65 ± 9	44 ± 5	31 ± 5	27 ± 4	22 ± 3
	Wingers	78 ± 14	52 ± 9	39 ± 8	33 ± 7	27 ± 6
High accelerations efforts (n.min^–1^)	Defenders	13.9 ± 2.0	10.5 ± 1.8	7.8 ± 1.3	6.7 ± 1.2	5.7 ± 1.0
	Pivots	11.8 ± 1.9	8.1 ± 1.2	5.9 ± 0.7	5.1 ± 0.6	4.3 ± 0.5
	Wingers	13.9 ± 2.3	9.9 ± 2.0	7.5 ± 1.5	6.5 ± 1.2	5.4 ± 0.9
High decelerations distance (m.min^–1^)	Defenders	66 ± 12	45 ± 7	33 ± 4	28 ± 4	23 ± 4
	Pivots	63 ± 13	42 ± 7	30 ± 5	24 ± 4	20 ± 3
	Wingers	69 ± 13	47 ± 9	34 ± 7	28 ± 6	24 ± 5
High decelerations efforts (n.min^–1^)	Defenders	14.4 ± 2.4	10.1 ± 2.1	7.5 ± 1.7	6.5 ± 1.4	5.4 ± 1.1
	Pivots	11.6 ± 2.0	8.0 ± 1.1	6.0 ± 0.7	5.0 ± 0.5	4.2 ± 0.5
	Wingers	13.7 ± 2.4	9.6 ± 1.7	7.2 ± 1.4	6.3 ± 1.2	5.2 ± 1.0

*Data presented as mean ± SD.*

*Number of players = 14 (4 Defenders, 2 Pivots, 8 Wingers); Number of observations = 143 (31 for Defenders, 27 for Pivots, 85 for Wingers); Number of games = 15.*

*HSR, high-speed running.*

**TABLE 2:** Fixed effects representing the effect of time window and the seasonal trend on all dependent variables for the three positional groups.

		Time window 30/300	Seasonal trend
Relative distance	Defenders	65 ± 6%;*ext*.large***	−3.2 ± 3.9%;*small*
	Pivots	64 ± 8%;*ext*.large***	−3.2 ± 3.9%;small*
	Wingers	65 ± 4%;*ext*.large***	2.3 ± 3.3%;*unclear*
Distance HSR	Defenders	483 ± 74%;*ext*.large***	12 ± 37%;*unclear*
	Pivots	505 ± 91%;*ext*.large***	2.6 ± 21%;*unclear*
	Wingers	402 ± 41%;*ext*.large***	−25 ± 13%;small**
HSR efforts	Defenders	377 ± 63%;*ext*.large***	31 ± 26%;moderate**
	Pivots	409 ± 84%;*ext*.large***	17 ± 23%;small*
	Wingers	329 ± 37%;*ext*.large***	−12 ± 15%;*small*
High accelerations distance	Defenders	166 ± 17%;*ext*.large***	3.9 ± 7.7%;*unclear*
	Pivots	196 ± 24%;*ext*.large***	14 ± 8%;moderate**
	Wingers	184 ± 12%;*ext*.large***	0.6 ± 5.4%;*unclear*
High accelerations efforts	Defenders	151 ± 12%;*ext*.large***	32 ± 12%;large***
	Pivots	171 ± 14%;*ext*.large***	13 ± 10%;moderate*
	Wingers	155 ± 7%;*ext*.large***	13 ± 9%;moderate**
High decelerations distance	Defenders	187 ± 17%;*ext*.large***	3.1 ± 9.7%;*unclear*
	Pivots	210 ± 23%;*ext*.large***	3.3 ± 9.7%;*unclear*
	Wingers	197 ± 11%;*ext*.large***	−6.5 ± 7.3%;small*
High decelerations efforts	Defenders	167 ± 16%;*ext*.large***	35 ± 15%;large***
	Pivots	171 ± 20%;*ext*.large***	16 ± 13%;moderate**
	Wingers	164 ± 10%;*ext*.large***	−1.0 ± 9.4%;*unclear*

*Data presented as percent effects ± 90% compatibility interval, with magnitude decision.*

*Time window 30/300 = predicted mean for the 30-s window minus the predicted mean for the 300-s window; Seasonal trend = predicted mean at the end of the season minus the predicted mean at the beginning of the season.*

HSR, high-speed running. * = Likely, ** = very likely, and *** = most likely substantial effect.

The analysis of the random effects showed clear, moderate-large individual differences in player means for all dependent variables, unclear differences in the slopes (i.e., the individual slopes representing the effect of the time windows), and clear small-moderate differences in game means (Table 3).

**TABLE 3:** Random effects (standard deviations) representing individual differences in player means and slopes, and differences in game means.

	Intercept	Time window 30/300	Date
Relative distance	5.7 ± 2.3%;large**	2.3 ± 3.2%;*unclear*	2.0 ± 0.9%;small**
Distance HSR	23 ± 10%;moderate**	4.5 ± 11%;*unclear*	11 ± 5%;small**
HSR efforts	19 ± 8%;moderate**	7.0 ± 12%;*unclear*	11 ± 5%;moderate**
High accelerations distance	12 ± 5%;large**	3.7 ± 5.4%;*unclear*	2.9 ± 1.8%;small*
High accelerations efforts	12 ± 5%;large**	−3.0 ± 3.2%;*unclear*	4.9 ± 2.1%;small**
High decelerations distance	11 ± 5%;large**	2.2 ± 5.0%;*unclear*	4.9 ± 2.1%;small**
High decelerations efforts	11 ± 5%;large**	2.1 ± 5.1%;*unclear*	6.4 ± 2.6%;moderate**

*Data presented as standard deviation ± 90% compatibility interval, with magnitude-based decision.*

*Intercept = individual differences in player true means; Time window 30/300 = predicted mean for the 30-s window minus the predicted mean for the 300-s window; Date = differences in game means after removing seasonal trend.*

HSR, high-speed running. * = Likely, ** = very likely.

## Discussion

The present study was designed to determine the positional differences along with the seasonal trend in the peak physical demands of elite futsal competition by quantifying the MDS of match play of different relative (per minute) external load variables between five different time windows in elite futsal competition. With respect to the initial objective of this study, it was found that peak physical demands during elite futsal competition are position-dependent for all time windows analyzed. Additionally, moderate-large individual differences in player means for all dependent variables and clear differences between games for most dependent variables were found. Another major finding of this study was that seasonal trends are also position and variable-dependent.

Our study highlights some interesting results related to both the fixed and random effects. There was a clear, extremely large effect of the time window for all dependent variables in all positional groups (Table 2). Interestingly, relative distance was the dependent variable with the smallest difference between the 30-s and 300-s window (∼65% for all groups), while the HSR distance (∼400-500%) and HSR efforts (∼330-410%) were the dependent variables with the largest differences. These results have important practical applications for training design, as they reflect the importance of knowing to what extent the different physical output targets must be adjusted in relation to the duration of a training drill. For example, for a same given drill performed either as 10 × 30-s repetitions or one whole 300-s exercise, the target for relative distance for the defenders would need to be ∼930 m in each 30-s repetition and ∼560 m for the 300-s drill. Conversely, the high-speed running distance would decrease from ∼235 efforts to ∼40.

Positional differences in the MDS of match play have been previously reported in different team-sports such as football (Hopkins et al., 2009), basketball (Vázquez-Guerrero et al., 2020), and rink hockey (Fernández et al., 2020). It is worth discussing that even though differences between playing position groups within each time window were not assessed due to the limited sample size, the results of this study suggest that peak physical demands are “higher” for defenders and wingers than for pivots for all variables analyzed. In accordance with these results, a previous study conducted by Serrano et al., 2020 reported similar patterns when describing differences across playing positions by evaluating the physical demands of official futsal matches through the traditional approach based on average values. It is possible to hypothesize that these differences may be attributed to the nature of each specific playing-position role with its particular technical and tactical requirements during the offensive (López, 2017; Méndez et al., 2019; Ohmuro et al., 2020) and the defensive phase of the game (López, 2017; Ohmuro et al., 2020). In contrast with the differences found with the pivots, our results showed similar values of the MDS in all dependent variables for defenders and wingers which could be explained by the continuous exchangeability that both playing-positions perform during games (Caetano et al., 2015; Serrano et al., 2020). This combination of findings provides some important support for training prescription, especially when attending to individualized training and rehabilitation programs and return-to-play protocols after injury (Vázquez-Guerrero et al., 2020).

To the best of the authors’ knowledge, this is the first study in elite futsal to compare the MDS of match play across different positions and time windows. However, comparisons of our results with those of other studies performed in other team sports (Martín-García et al., 2018; Salazar and Castellano, 2019; Fernández et al., 2020; Vázquez-Guerrero et al., 2020) reveal some interesting findings. During the MDS of match play, futsal players cover less distance than football players regardless of the position for the 60-, 180-, and 300-s time windows (Martín-García et al., 2018) but more than basketball players (Salazar and Castellano, 2019; Vázquez-Guerrero et al., 2020) only for the 180- and 300-s time windows. A possible explanation for these differences could be the larger playing area of the football field (100 m long and 80 m wide) compared to the futsal and the basketball courts (40 and 28 m long; 20 and 15 m wide respectively). Besides, futsal players cover a lower amount of HSR distance during the MDS of match play than rink hockey players (Fernández et al., 2020) but greater than basketball players in all positions and time windows (Vázquez-Guerrero et al., 2020). It seems important to recall the fact that rink hockey players do not run but rather skate, which may explain the large difference found in these results. Another interesting finding is that futsal players cover higher volume of high intensity acceleration and deceleration distance and perform a greater number of high intensity accelerations and decelerations than basketball players for all positions and time windows. The reason for such differences is not clear but it may be related to the idiosyncrasy of the short stoppages and interruptions during futsal matches (i.e., outsides, corner-kicks) in which players continue to move even when the ball is not in play (Illa et al., 2020).

The results regarding the seasonal trends also have important practical applications. While most trends were unclear or only likely substantial and small in magnitude, some clear moderate-large trends were identified. For example, defenders were the only positional group to report clear moderate-large positive trends for HSR efforts, high-acceleration efforts, and high-deceleration efforts. On the other side, wingers reported small decrements in HSR distance. These results may be influenced by contextual factors such as the strength of the opposition; however, this cannot be speculated upon without further investigation.

As mentioned in the results section, the analysis of the random effects returned clear, moderate-large individual differences in player means for all dependent variables; this signifies that independent of the playing position, players differ overall in how “demanding” the MDS are for all variables. This information, in conjunction with the information regarding the positional differences and the seasonal trends, can assist the practitioner to further individualize the training prescription. Similarly, clear differences were found between games for most dependent variables; this difference again points to the likely influence of contextual factors on the MDS, but it also provides practical information to further modify the game preparation. Further research should be undertaken to investigate the influence of the contextual factors in the magnitude of the match-to-match variability of the MDS of match play in elite futsal competition.

## Conclusion

One of the most significant findings to emerge from this study is the extremely large effect of the time window for all dependent variables in all positional groups. This study has also identified clear differences between games for most dependent variables and moderate-large individual differences in player means for all dependent variables alongside with clear moderate-large positive trends for high-speed running (>18 km⋅h^–1^) efforts, high-acceleration efforts, and high-deceleration efforts for defenders. With these results, practitioners are provided with some insight regarding to the MDS of match play which may lead to a more precise position-specific training prescription. Evidently peak physical demands of elite futsal competition are very high, and therefore, players’ training program prescription should be properly designed to prepare players for such high demanding exposures. However, practitioners are advised to consider individual variability when attending to positional requirements and adapting these scenarios to the duration of the training drills.

## Data Availability Statement

The raw data supporting the conclusions of this article will be made available by the authors, without undue reservation.

## Ethics Statement

The studies involving human participants were reviewed and approved by the Comitè d’Ètica d’Investigacions Clíniques de l’Administració Esportiva de Catalunya. The patients/participants provided their written informed consent to participate in this study.

## Author Contributions

JI contributed to the conception and design of the study, contributed to the data collection and interpretation of results, and contributed to the manuscript writing. DF participated in the design of the study, contributed to the data reduction/analysis and interpretation of results, and contributed to the manuscript writing. FS participated in the design of the study, contributed to the data reduction/analysis and interpretation of results, and contributed to the manuscript writing. All authors have read and approved the final version of the manuscript and agree with the order of presentation of the authors.

## Conflict of Interest

The authors declare that the research was conducted in the absence of any commercial or financial relationships that could be construed as a potential conflict of interest.

## Abbreviations

UWB: ultra-wideband
MDS: most demanding scenarios
LNFS: Liga Nacional de Fútbol Sala
UEFA: Union of European Football Associations
HSR: high-speed running.
